# Cryobiopsy for Secondary Pulmonary Alveolar Proteinosis

**DOI:** 10.7759/cureus.60530

**Published:** 2024-05-17

**Authors:** Hiroshi Kobe, Nobuyoshi Hamao, Takashi Niwa, Tadashi Ishida

**Affiliations:** 1 Department of Respiratory Medicine, Ohara Healthcare Foundation, Kurashiki Central Hospital, Kurashiki, JPN; 2 Department of Respiratory Medicine, Kanagawa Cardiovascular and Respiratory Center, Yokohama, JPN

**Keywords:** interstitial lung disease, cryobiopsy, follicular lymphoma, granulocyte-macrophage colony-stimulating factor, pulmonary alveolar proteinosis

## Abstract

Secondary pulmonary alveolar proteinosis (SPAP) is one of the diffuse parenchymal lung diseases, and the utility and safety of transbronchial lung cryobiopsy (TBLC) for diagnosing SPAP are unknown. A case of SPAP diagnosed by TBLC is presented. Specimens that were useful for diagnosis were collected, and there was no adverse event following TBLC. The usefulness of TBLC for interstitial lung disease has been widely reported, but there are few reports of SPAP. We present the clinical course of TBLC in the diagnosis of SPAP.

## Introduction

Pulmonary alveolar proteinosis (PAP) is one of the diffuse parenchymal lung diseases characterized by the accumulation of lipoproteinaceous material in alveolar regions due to abnormal surfactant homeostasis of alveolar macrophages [1]. Secondary PAP (SPAP) accounts for 5-10% of adult PAP cases caused by hemopoietic disorders, immune dysregulation, infections, inhalation, lysinuric protein intolerance, and drug-induced or iatrogenic. 25-53% of SPAP cases require video-assisted thoracoscopic surgery (VATS) or open lung biopsy because bronchoalveolar lavage (BAL) and transbronchial lung biopsy (TBLB) are not sufficient for a definitive diagnosis [2]. BAL fluid typically displays a characteristic milky appearance. Lung histopathology typically shows diffuse, dense acellular eosinophilic material in the airways. Transbronchial lung cryobiopsy (TBLC) is when compressed gas released at high flow rates expands rapidly to create ultra-low temperatures, allowing large tissue samples to be taken in a freeze-thaw cycle [3]. TBLC is a diagnostic tool that shows high levels of agreement with surgical lung biopsy (SLB) in diagnosing interstitial lung disease (ILD) [4]. The utility and safety of TBLC for ILD other than SPAP are established, but they have been unclear for SPAP. A case of suspected ILD in which TBLC was performed and later diagnosed as SPAP is presented. Following TBLC, no apparent adverse events occurred, and good-quality specimens were obtained that contributed to the diagnosis. TBLC may be an effective biopsy method for patients with SPAP who previously required VATS.

## Case presentation

**Figure 1:**
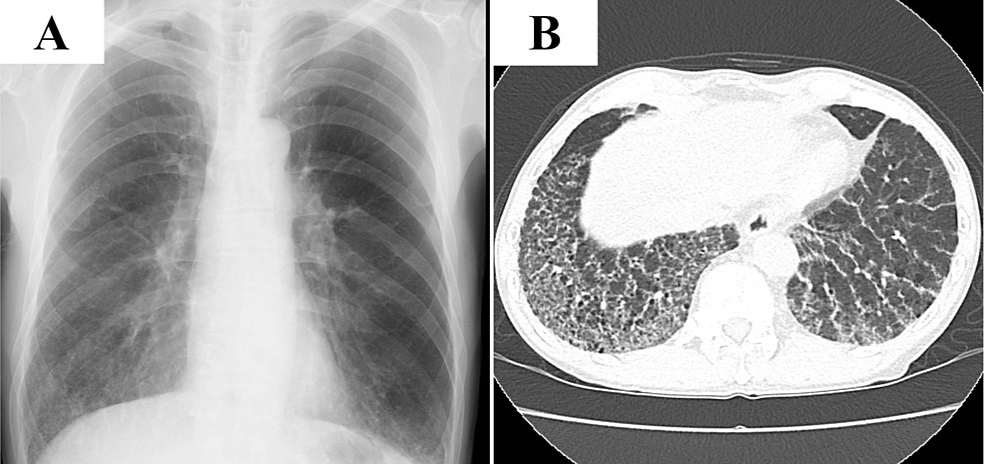
Chest X-ray and chest high-resolution computed tomography at initial examination. A: Chest X-ray showed ground-glass opacity in the lower lung field. B: Chest high-resolution computed tomography: reticular and ground-gross shadows extend along the bronchovascular bundles and just below the pleura in the lower lobes of both lungs.

A 66-year-old Japanese man with untreated stable follicular lymphoma was referred from the hematology clinic for investigation of abnormal findings detected incidentally. He smoked for 37 years and quit smoking when he was 57 years old. He worked in a granite mine for about 10 years and had been exposed to dust, granite, and iron. He had no symptoms of breathlessness at all. The chest X-ray showed ground-glass opacity in the lower lung field, and the chest CT showed reticular and ground-gross shadows extending along the bronchovascular bundles and just below the pleura in the lower lobes of both lungs (Figures 1A, 1B).

Hospitalized for bronchoscopy due to suspected infection or pulmonary infiltration of lymphoma. On admission, his vital signs were a blood pressure of 153/96 mmHg, a temperature of 36.1°C, a pulse rate of 91 beats/min, a respiratory rate of 16 breaths/min, and a peripheral oxygen saturation of 96% in ambient air. Laboratory findings are presented in Table 1.

**Table 1:** Laboratory findings

Hematology and biochemistry	Patient's value	Normal value
White blood cells (WBC)	3700	3300-8600/μL
Neutrophils	50.2	38.0-77.0 %
Lymphocytes	36.9	15.0-53.0 %
Monocytes	9.7	0-13.0 %
Eosinophils	1.9	0-6.0 %
Basophils	1.3	0-2.0 %
Hemoglobin (HB)	14.2	13.7-16.8 g/dL
Platelets (PLT)	22.7×10^4^	16.0-36.0×10^4 ^/μL
Total protein (TP)	7.8	6.6-8.1 g/dL
Albumin (ALB)	3.5	4.1-5.1g/dL
Aspartate aminotransferase (AST)	208	13-30U/L
Alanine aminotransferase (ALT)	47	10-42U/L
Lactate dehydrogenase (LDH)	334	124-222U/L
Blood urea nitrogen (BUN)	7	8-20mg/dL
Creatinine (CRE)	0.72	0.65-1.07mg/dL
Sodium (Na)	140	138-145mmol/L
Potassium (K)	4.3	3.6-4.8mmol/L
C-reactive protein (CRP)	0.02	0-0.14mg/dL
Krebs von den Lungen-6 (KL-6)	8071	105-401U/mL
Surfactant protein-D (SP-D)	2360	<110ng/mL
Antinuclear antibody (ANA)	80 times	<40 times
Anti-neutrophil cytoplasmic antibody (ANCA)	negative	negative
anti-cyclic citrullinated peptide (CCP)	<0.5	<4.5U/mL
anti-aminoacyl tRNA synthetase (ARS)	<5.0	<25.0

Arterial blood gas analysis showed that the partial pressure of arterial oxygen was 82.0 mmHg in ambient air (reference range, 80.0-100.0 mmHg). The following results were pulmonary function tests, forced vital capacity (FVC); 4.65 L (predicted value, 3.64 L), %FVC; 126.1%, forced expiratory volume in one second (FEV_1.0_); 3.82L (predicted value, 2.98 L), % FEV1.0; 128.6%, lung carbon monoxide diffusing capacity (DL_CO_); 13.94 mL/min/mmHg (predicted value, 15.93 mL/min/mmHg), and %DL_CO_; 92.9%.

**Figure 2:**
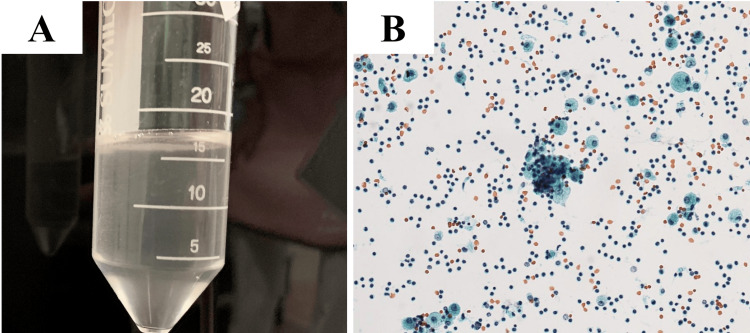
Bronchoalveolar lavage fluid appearance and cytology. A: Bronchoalveolar lavage fluid appearance: mildly cloudy. B: Cytology of bronchoalveolar lavage fluid: foamy macrophages are observed, x20.

BAL fluid (right lower lobe B_8_) was grossly mildly cloudy (Figure 2A), and cell counts were 1.2 x 10^6^/ml with 1% neutrophil, 85% lymphocyte, and 14% macrophage. Foamy macrophages were seen on cytology (Figure 2B). No significant bacteria were cultured in the BAL fluid.

**Figure 3:**
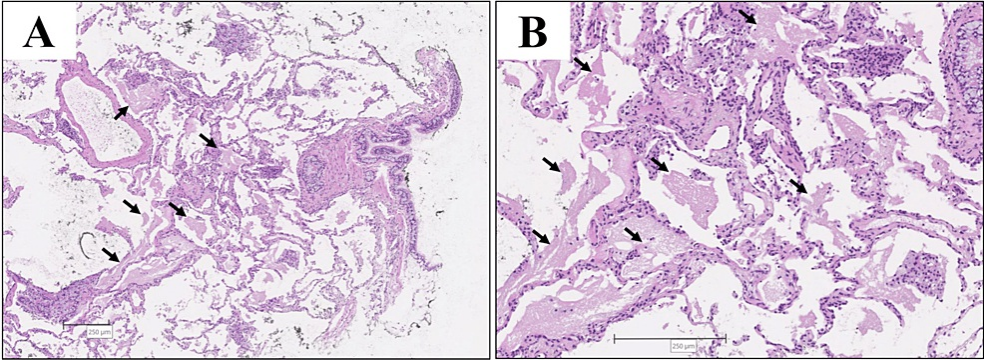
Lung tissue obtained by transbronchial lung cryobiopsy Transbronchial lung cryobiopsy: hematoxylin and eosin stain. (A) x4, (B) x10. Acidophilic granular structures were seen in the alveolar region (black arrows)

TBLC was performed by a 2.4-mm cryoprobe in the right lower lobe B9, twice times for biopsy, and the freezing time was five seconds each. Balloon occlusion was used for bleeding management, and no other additional procedures were performed for bleeding. Lung tissue, hematoxylin, and eosin stain revealed acidophilic granular structures in the alveolar region (Figure 3).

**Figure 4:**
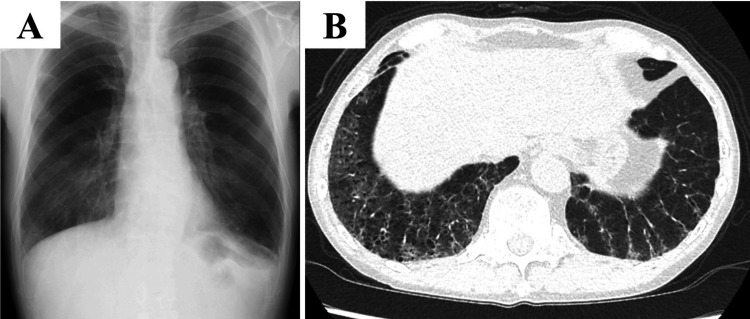
Chest X-ray and chest high-resolution computed tomography at a year after diagnosis A: On the chest X-ray, ground-glass opacity was improved. B: Chest high-resolution computed tomography, ground-glass opacity with interlobular septal was improved.

There were no complications associated with TBLC. No findings suggested pulmonary infiltration of lymphoma or infection. Based on the above, PAP was diagnosed. Serum granulocyte-macrophage colony-stimulating factor autoantibody was negative, and he was diagnosed with PAP secondary to lymphoma. Since the severity of SPAP was mild, he continued to be followed up without treatment. One year has now passed since SPAP diagnosis; his lymphoma has not worsened, and SPAP has spontaneously lightened (Figure 4).

## Discussion

SPAP is commonly caused by hemopoietic disorders, immune dysregulation, infections, inhalation, lysinuric protein intolerance, and drug-induced or iatrogenic factors and is usually negative for serum granulocyte-macrophage colony-stimulating factor autoantibody [1,2]. Relying solely on serological tests to establish a diagnosis can be challenging, making it essential to obtain a definitive diagnosis to understand the underlying disease.

H. Ishii et al. reported that, among 40 patients with SPAP, a definitive diagnosis of SPAP was made using BAL in 21 cases, TBLB in nine cases, and VATS in 10 cases [2]. For some patients, BAL and TBLB are not sufficient for a definitive diagnosis of SPAP. Until now, SLB was the only option when BAL and TBLB were inadequate. It has been reported in the field of ILD that TBLC is less invasive than SLB and has a higher diagnostic yield than TBLB [4-6]. It is expected that more PAP cases could be diagnosed by performing TBLC in cases in which SLB cannot be performed.

Bleeding and pneumothorax are the common complications of TBLC [7]. According to a recent systematic review, TBLC for ILD had a higher bleeding risk than TBLB [8]. No reports of elevated bleeding or pneumothorax incidence could be found in patients with SPAP. In addition, a higher risk of infection after TBLC in patients with PAP is a concern because patients with PAP are susceptible to infections due to reduced neutrophil and macrophage function in the alveoli [9]. Inoue Y. et al. reported that 5.7% of patients with PAP in their Japanese cohort developed infections regardless of bronchoscopic procedures [10]. Azuma K. et al. reported that no patients with PAP developed fever after bronchoscopy, though 53.5% of patients with PAP were prophylactically prescribed oral antibiotics after bronchoscopy [11]. As noted earlier, the incidence of infection after TBLB is not high, but there are no such reports regarding TBLC. As neither pneumothorax, severe bleeding, nor infection were observed in the present case, we consider it feasible to perform TBLC in patients with SPAP.

## Conclusions

We reported the case of TBLC for SPAP. SPAP is often challenging to diagnose due to the frequent absence of serum granulocyte-macrophage colony-stimulating factor autoantibody, and BAL and TBLB are often not enough to make the diagnosis. TBLC can be a valuable method to improve diagnostic accuracy in suspected cases. Although patients with PAP are known to have a higher risk of respiratory infections, TBLC may be feasible if conducted with adequate precautions. Even when SPAP is definitively diagnosed, treatment may not always be necessary. It is important to carefully monitor the progression of SPAP. Making a definitive diagnosis contributes to determining an appropriate treatment strategy.
